# The effectiveness of *Rutin* for prevention of surgical induced endometriosis development in a rat model

**DOI:** 10.1038/s41598-021-86586-4

**Published:** 2021-03-30

**Authors:** Hatef Talebi, Mohammad Reza Farahpour, Hamed Hamishehkar

**Affiliations:** 1 Department of Basic Sciences, Faculty of Veterinary Medicine, Urmia Branch, Islamic Azad University, Urmia, Iran; 2 Department of Clinical Sciences, Faculty of Veterinary Medicine, Urmia Branch, Islamic Azad University, Urmia, Iran; 3 Drug Applied Research Center, Tabriz University of Medical Sciences, Tabriz, Iran

**Keywords:** Molecular biology, Health care, Medical research

## Abstract

Apoptosis and antioxidant mechanisms are pathways for the treatment of endometriosis (Endo). Rutin (Rtn) is an antioxidant flavonol that induces apoptosis. This study, for first time, was conducted to evaluate the effects of rutin on Endo through apoptosis and antioxidant mechanisms. The experimental Endo was induced in 24 rats and then the animals were subdivided into Endo-sole, 3000 and 6000 µg/kg rutin (Rtn-3000 and Rtn-6000) and vitamin C groups. After 4 weeks, the expression of Bcl2, Bax, anti Pro Caspase-9, cleaved Caspase-9, pro PARP, pro Cleaved PARP, Pro PARP, pro mTOR and mTOR were assessed by western blotting technique. The protein concentrations of malondialdehyde (MDA), total antioxidant capacity, and super oxide dismutase and gutathione peroxidase were also evaluated. TUNEL staining was also used for the detection of apoptosis. Caspase-9 and concentration of antioxidants were higher in the treated groups compared to Endo-sole group (*P* < 0.05). The results also showed that rutin decreased the expression of Bcl2 and MDA concentration (*P* < 0.05). The results for TUNEL staining showed that the animals treated with Rtn-6000 and vitamin C showed higher apoptosis. Rutin induces apoptosis by the expression of Bcl-2, Bax and caspase and also antioxidant activity by increasing antioxidants concentrations.

## Introduction

Endometriosis (Endo) is one of the most usual causes for chronic pelvic pain that is known with endometrial tissue outside the uterine cavity^1^. It is commonly known with signs such as chronic pelvic pain, infertility, menstrual irregularity, dyspareunia and loss quality of life^2^. The pathophysiology and etiology of the Endo is still unknown. It needs estrogen-dependent condition, but its symptoms are cyclic^3^. Similar to eutopic endometrium, ectopic endometrium induces responses versus hormonal changes through inducing pain and inflammation in the target sites^4^. Apoptosis has an important role in keeping tissue homeostasis by eliminating excess and dysfunctional cells. Apoptosis mechanism has received much attentions in patients with eutopic and ectopic endometrium. Mitochondrial apoptosis plays important role in cell apoptosis. Among proteins involved in cell apoptosis, Bcl-2 family proteins especially B-cell lymphoma protein 2 (Bcl-2)-associated X (Bax) protein play important role in apoptosis, because these participate in mitochondrial apoptosis by preventing and promoting apoptosis^5^. The Bax protein induces the cascade of reactions through releasing cytochrome c from mitochondria and causes cell death. Bcl-2 inhibits Bax activity and blocks activation of apoptotic machinery^6^. The PI3K/mTOR pathway is also activated in ovarian Endo^7^. Poly ADP-ribose polymerase-1 (PARP-1) is an important protein that repairs DNA single strand break (SSB)^8^. Faulted SSB repair accumulates of double strand breaks (DSB) and are then repaired by DSB repair mechanisms^9^. Mammalian Target of Rapamycin (mTOR) activation and its up-regulation are related to proliferation and biological aggressiveness in some tumors^8^. Caspases are a family of endoproteases that play important role in regulating programmed cell death. Caspase-9 activation is a response for apoptotic stimuli and is started with the mitochondrial release of cytochrome c^10^. On the other hand, reactive oxygen species (ROS) play important role in reproductive system such as Endo and infertility, so that oxidative stress increases Endo^11^.

Different agents are used for the prevention, the management and the treatment of Endo. Natural agents such as medicinal plants and their derivations have commonly been used. Rutin (quercetin-3-rhamnosyl-glucoside) is a flavonol that is significantly found in some fruits and vegetables^12, 13^. It has some pharmacological activities, such as antibacterial^14^, immunomodulatory, antioxidant, and neuroprotective activities^15^. Rutin protects reproductive system against oxidative stress in diabetes mellitus^16^. It shows anticancer activity by preventing cell proliferation, reducing glutathione (GSH) and inducing apoptosis in cancer cells^17, 18^. Rutin induces apoptosis and has antioxidant activity that can be profitable in the treatment of the Endo and it is used as a novel agent. This study was conducted to evaluate the effects of rutin in a rat model with surgically induced Endo, by evaluating the role of Bcl2, Cleaved caspase, Cleaved PARP and mTOR expression.

## Results

### The effect of Rtn on protein expressions

**Figure 1:**
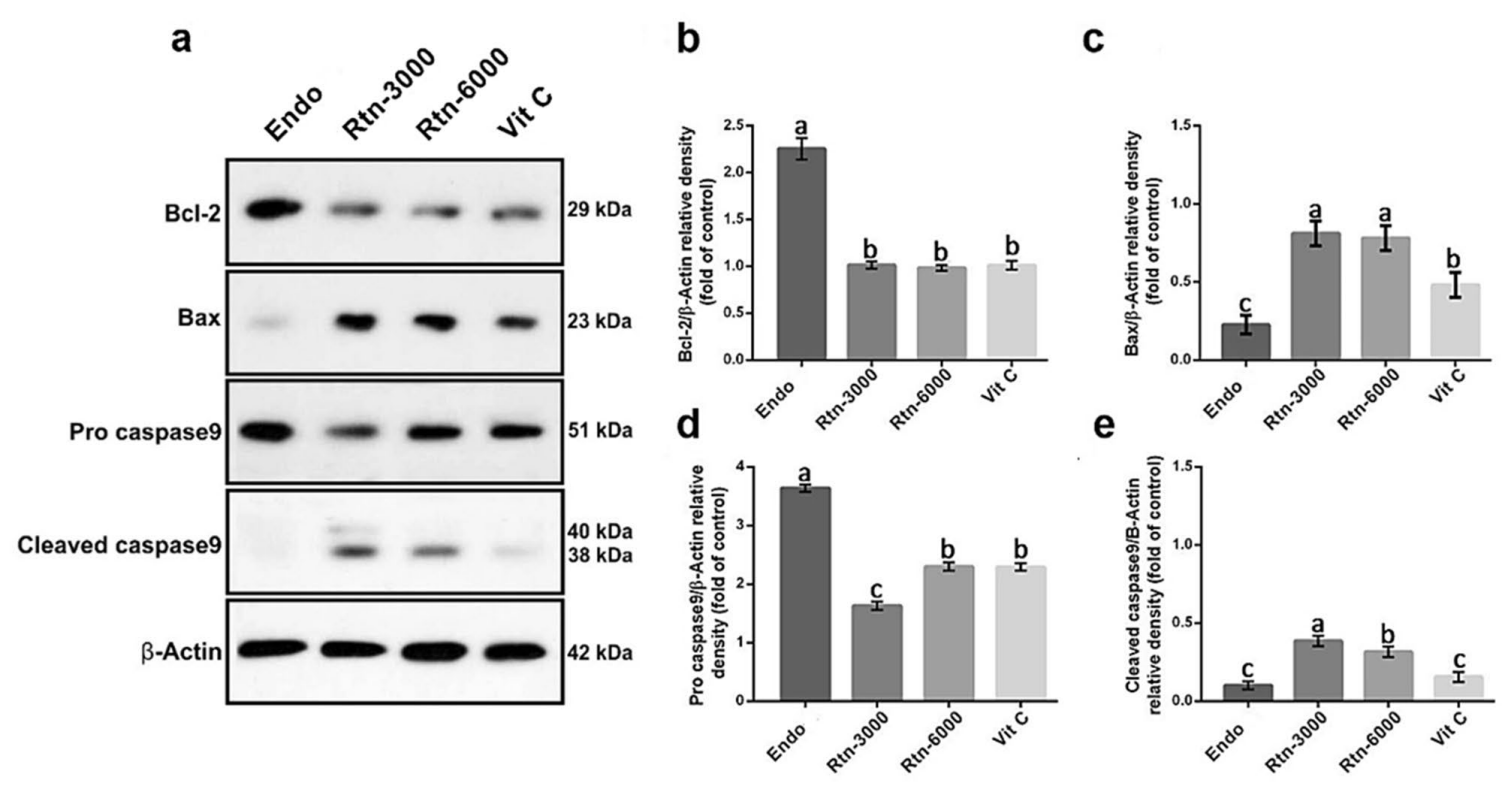
(**a**) Determination of Bcl-2, Bax, Pro caspase9, Cleaved caspase9 and β-actin by Western blot in cell lysates. Western blotting of Bcl-2, Bax, Pro caspase9, and Cleaved caspase9 were performed on lysates of the various cell lines by specific antibodies. β actin was used as an internal control. (**b**), (**c**), (**d**) and (**e**), Quantitative analyses of Bcl-2, Bax, Pro caspase9, and Cleaved caspase9 were performed in the various cell lines by Western blot analysis. The mean optical density ratio for Bcl-2, Bax, Pro caspase9, and Cleaved caspase9 was calculated in per endometriotic cell type and compared to control endometrial cells. Superscripts (a, b, c) show significant differences between groups at *P* < 0.05.

The results for western blotting of Bcl-2, Bax, Pro caspase9 and Cleaved caspase9 are shown in Fig. 1. The results showed that the animals in Endo-sole group showed higher expressions for Bcl-2 and Pro caspase9 compared to other groups (*P* < 0.05). However, the administration of Rtn in levels of 3000 and 6000 decreased the expressions of Bcl-2 and Pro caspase9 compared to control group (*P* < 0.05). With regards to golden standard (Vit C), the animals in Rtn-6000 group did not show significant difference for expression of Bcl-2 and Pro caspase9 (*P* > 0.05). However, the animals in Rtn-3000 group showed lower expression for Pro caspase9 compared to Rtn-6000 and Vit C (*P* < 0.05). The results for the expression of Bax and Cleaved caspase9 showed that the rats administrated with rutin showed higher expression compared to Endo-sole group (*P* < 0.05). The results did not show significant difference between the levels of rutin for the expression of Bax (*P* > 0.05), but the animals treated with rutin in lower dose (Rtn-3000) showed higher expression for Cleaved caspase9 compared to higher dose (Rtn-6000) (*P* < 0.05). Considering Vit C, the administration of rutin increased expression of Bax and Cleaved caspase9 (*P* < 0.05).

**Figure 2:**
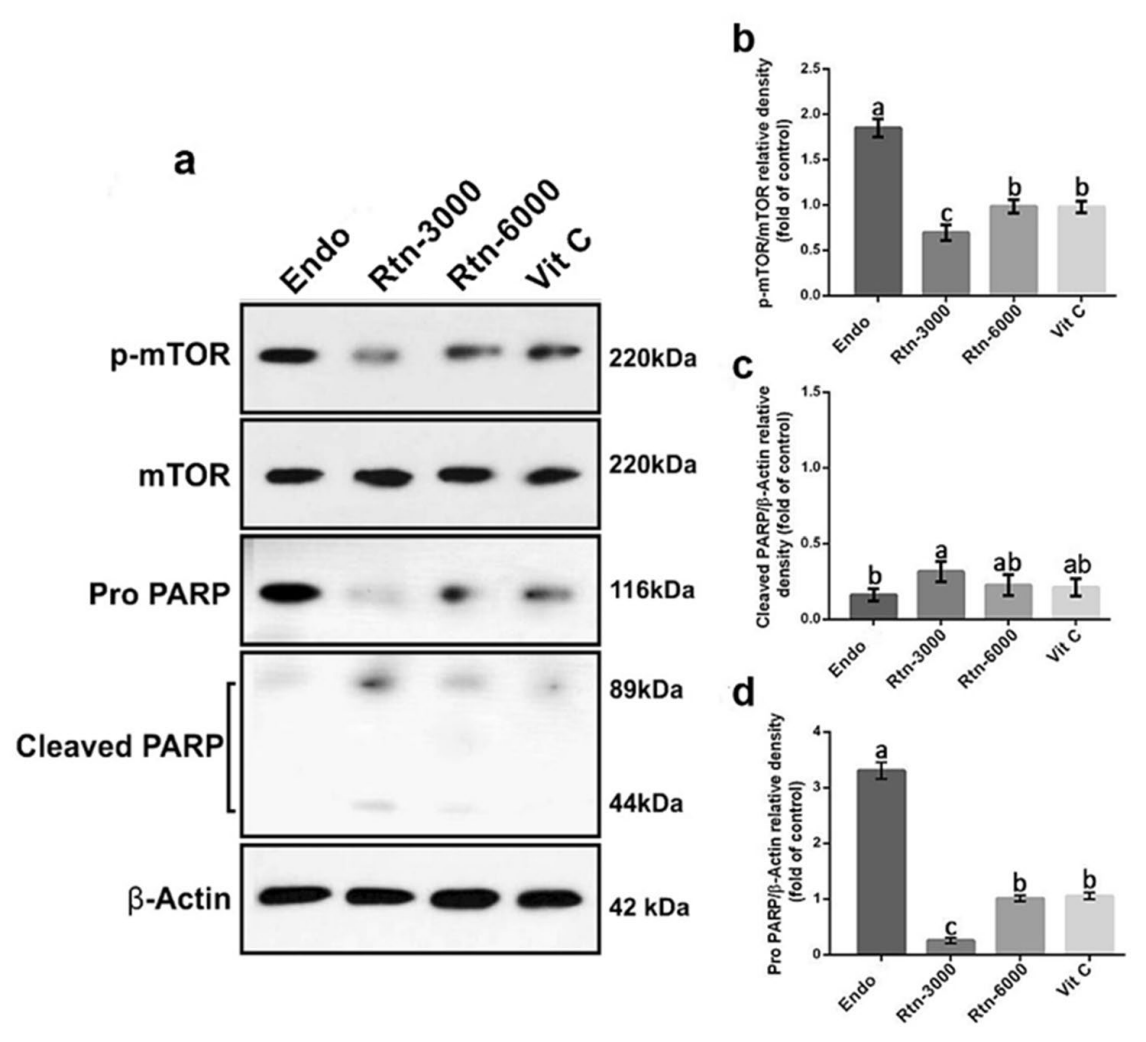
(**a**) Determination of p-mTor, mTOR, Pro PARP, Cleaved PARP and β-actin by Western blot in cell lysates. Western blotting of p-mTOR, mTOR, Pro PARP and Cleaved PARP were performed on lysates of the various cell lines by specific antibodies. β actin was used as an internal control. (**b**), (**c**) and (**d**), Quantitative analyses of p-mTOR, mTOR, Pro PARP and Cleaved PARP were performed in the various cell lines by Western blot analysis. The mean optical density ratio for p-mTor, mTOR, Pro PARP and Cleaved PARP was calculated in per endometriotic cell type and compared to control endometrial cells. Superscripts (a, b, c) show significant differences between groups at *P* < 0.05.

Our findings for p-mTOR, mTOR, Pro PARP and Cleaved PARP by western blotting are shown in Fig. 2. The results showed that rats in Endo-sole group showed higher expression for p-mTOR and Pro- PARP compared to other groups (*P* < 0.05). The results showed that expressions of p-mTOR and Pro-PARP were significantly lower in animals in Rtn-3000 group compared to other group (*P* < 0.05). It was no observed significant differences between Vit C and Rtn-6000 (*P* > 0.05) for expressions of p-mTOR and Pro-PARP (*P* > 0.05). The results for Cleaved PARP did not show significant difference between Endo-sole group, Rtn-6000 and Vit C groups (*P* < 0.05). The results showed significant difference between Rtn-3000 group with Endo-sole and Vit C groups (*P* < 0.05).

### Rtn enhanced apoptosis ratio

**Figure 3:**
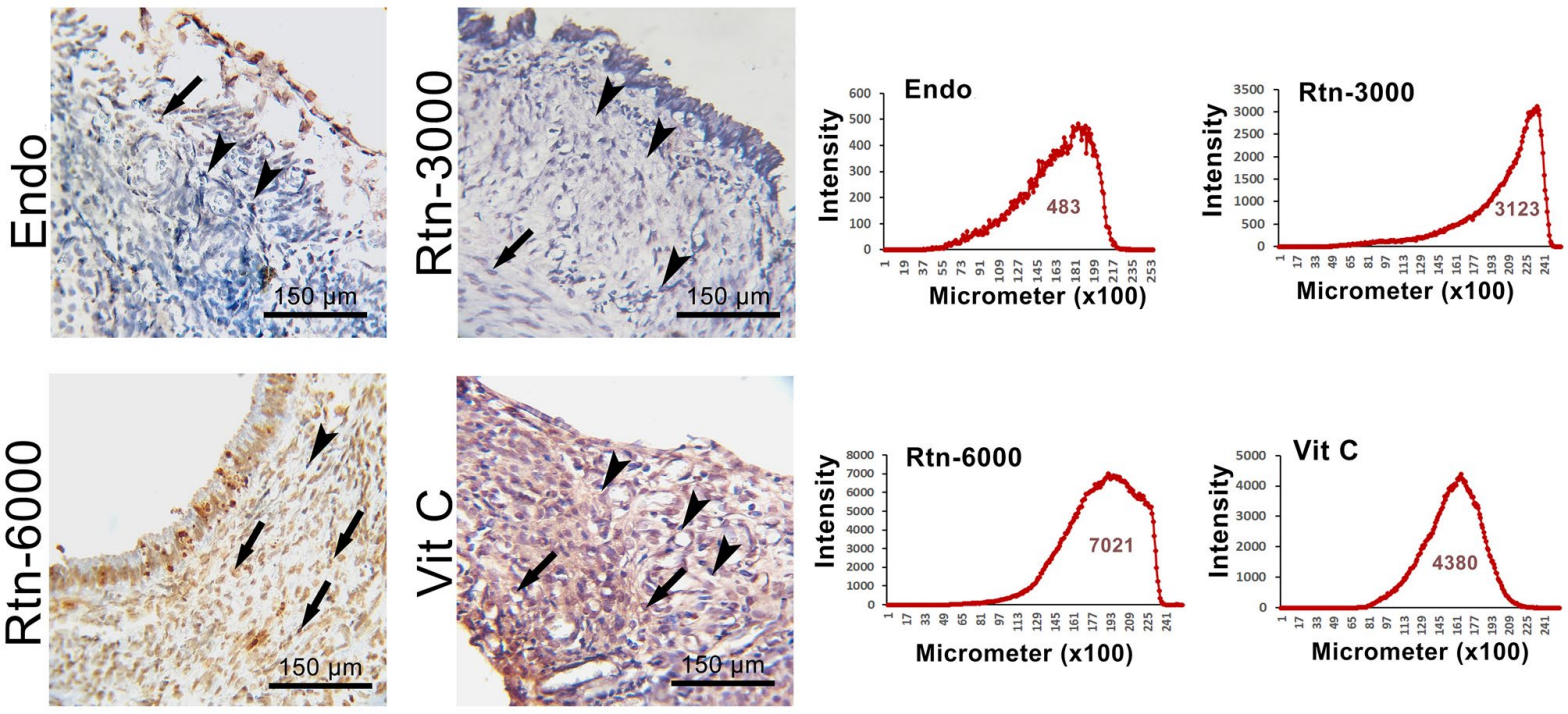
TUNEL staining of ectopic endometrial tissue. Note increased apoptotic cells in vitamin C-treated and Rtn-6000-treated groups compared to control and other experimental groups. The software analyses of the pixel-based intensity of brown reaction (representing TUNEL reaction) in 25,200 × 25,200 µm of tissue in different groups are presented. As shown, the section from the control group represents the lowest and the section from Rtn-6000 and Vit C are representing the highest TUNEL reactions, respectively.

The results for TUNEL staining in Fig. 3 showed higher apoptosis in animals treated with Rtn-6000 and Vit C. It means that Rtn-6000 and Vit C significantly induce apoptosis. The results showed that staining intensity was significantly higher in Rtn-6000 and Vit C groups.

### Biochemical estimations

**Figure 4:**
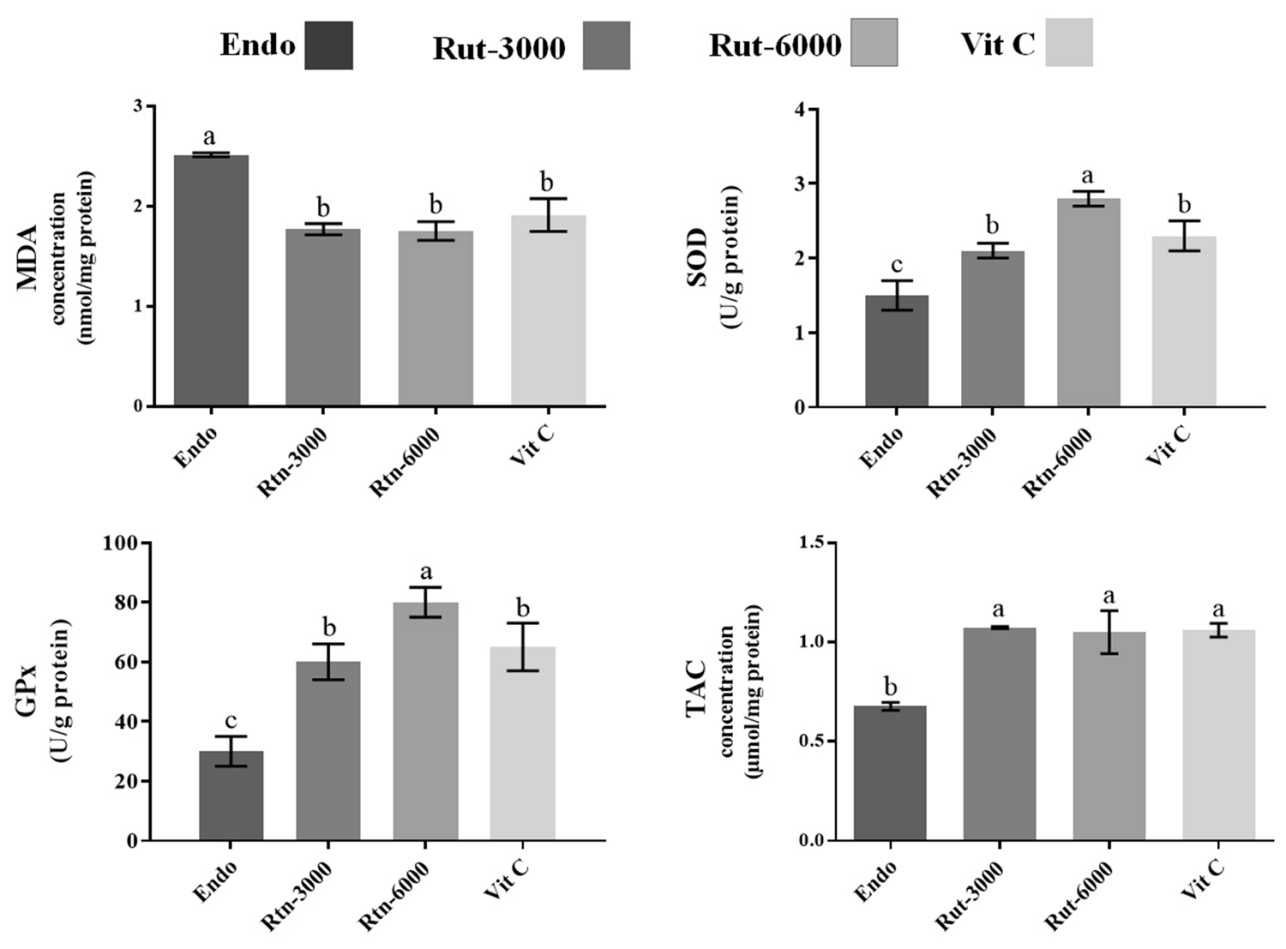
Determination levels of MDA, SOD and GPx and TAC in the different cells. Superscripts (a, b) show significant differences between groups at *P* < 0.05.

The results for MDA, SOD and GPx and TAC concentrations are shown in Fig. 4. The results showed that the concentration of MDA was significantly lower in Endo-sole group compared to other groups, respectively (*P* < 0.05). There was not significant difference between the animals in Vit C group and other treated animals for MDA and TAC (*P* < *0.05*). However, the rats treated with higher level of rutin (6000) showed higher levels for GPx and SOD.

## Discussion

The pathophysiology of the Endo is not yet known and the treatment of Endo is controversial. Different agents are used for the treatment of Endo. Some studies have shown that antioxidants might be beneficial for the treatment of Endo^19, 20^. In this study, for the first time, rutin, as an antioxidant agent and also inducer apoptosis, was used in a rat model of Endo.

Bcl-2 family proteins regulate mitochondria-dependent pathway for apoptosis. It was reported that Bcl-2 up-regulation can inhibit programmed cell death and hasten the progression of Endo and finally result disease pathogenesis via cytokine production^21^. Studies have suggested that up-regulation of Bax and down-regulation of Bcl-2 increase apoptosis^22, 23^. In the present study, increased the expression of Bax and decreased the expression of Bcl-2 were observed in animals treated with rutin compared to animals in Endo-sole group. It means that rutin induces apoptosis and TUNEL staining confirm our findings for induction of apoptosis in rutin groups. It means that groups, especially Rtn-6000 and Vit C induce apoptosis. Parallel to our findings, previous studies have reported that rutin induces apoptosis in cancer cells^17, 18^. Other study showed that rutin decreases cisplatin-induced reproductive damage by inducing apoptosis in male rats^24^. The results also showed that rutin increased the expression of Cleaved caspase, but no pro caspase, compared to animals in Endo-sole group. Caspases are a family of endoproteases that play important role in regulating programmed cell death. It was reported that ESR2 destroys apoptosome production via interacting and preventing the activation of caspase 9 in endometriotic lesions^25^. The results show that rutin induces apoptosis via regulation of Bcl-2, Bax and caspase 9.

The results also showed decreased the expression of pro mTOR in the treated animals compared to Endo-sole group. It was no observed significant difference between groups for the expression of mTOR. This gene has an essential role in angiogenesis and growth of endometriotic implants that hastens Endo^26^. It was reported that the administration of temsirolimus blockers reduces Endo implants growth^27^. The results showed that rutin down-regulates the expression of mTOR. It means that rutin cannot prevent growth the Endo via the expression of mTOR. The expression of Cleaved PARP was significantly higher in Rtn-3000 compared to Endo-sole group. A study reported that inhibition of PARP causes lack of correction of SSBs in S phase and the errors cause to stalling and collapse of the replication forks that result in the persistence of DNA DSBs^9^. Seemingly, it does act in Endo through influencing on PARP.

The results also showed that the concentration of MDA was significantly lower, but SOD, GPx and TAC were higher in the treated animals compared to Endo-control group. Increased the levels of rutin did not have significant effects on concentration of MDA and TAC, but concentration of SOD and GPx were higher in dose of 6000 µg/kg. The increase in production of free radicals and decreased antioxidant potential induce oxidative stress. Increased oxidative stress was associated to Endo. Redox levels might be involved in the severity and the dynamics of Endo and its progression^11^. Parallel to our findings, Gupta et al.,^28^ showed that MDA and TAC is respectively higher and lower in animal model of Endo. Imbalance between ROS and antioxidant mechanisms cause follicular fluid, and ovary surrounding that causes the infertility status related to Endo^11^. Rutin protects reproduction system from oxidative stress in diabetes mellitus^16^. Thus, rutin decreases MDA levels by increasing antioxidants. Seemingly, rutin increases levels of antioxidants and reduces MDA and decreases Endo.

This study was conducted to evaluate the effects of Rtn on expression of Bcl2, Bax, Cleaved caspase, Cleaved PARP and mTOR expression in a rat model with surgically induced Endo. The results showed that rutin induces early apoptosis by the expression of Bcl-2, Bax and caspase and TUNEL staining confirmed the results. It also showed antioxidant properties that decreases Endo. It can be stated that rutin (especially 6000 µg/kg) improves Endo by apoptosis and antioxidant mechanisms.

## Materials and methods

### Materials

Rutin (Cat No: S0292 SIGMA) was prepared from Sigma Chemical Co. (St. Louis, MO, USA). Antibodies included anti Bcl2 (sc-492), Bax (sc-7480), PARP-1 (sc-8007), mTOR (sc-517464), p-mTOR Antibody (sc-293133) and β-Actin (C4): sc-47778) prepared from Santa Cruz Biotechnology’s. Moreover, Caspase-9 (Mouse mAb) Antibody prepared from Cell Signaling Technology.

### Animal model

In the current study, 24 female Wistar albino rats non-pregnant and null gravid were purchased from Pasture Institute (Amol-Iran). The animals were 10–12 weeks of age and had initial weight of 180 ± 20 g. The animals were kept under controlled condition in a room temperature of 21 ± 2 °C, 60 ± 5% humidity and 12 h light/dark cycles. All the used protocols were in agreement with ARRIVE guidelines such as study design, sample size, randomization, outcome measures, data analysis, experimental procedures, reporting the results, etc., in this study were approved by the Committee on the Ethics of Animal Experiments of Veterinary Faculty and the Islamic Azad University Council on Animal Care, Urmia, Iran (IAUIAC permit number: FW2019-10,370,501,972,043), in compliance with the Guide for the Care and Use of Laboratory Animals published by the US National Institutes of Health (NIH publication no.85–23, revised 1996). We declare that all methods were performed in accordance with the relevant guidelines and regulations.

### Induction of Endo

Endo was induced by transplanting the bowel mesentery as reported by Bilotas^29^. Summary, anesthetization was induced by intraperitoneal administration of ketamine (100 mg/kg) and 2% xylazine (40 mg/kg). After conducting laparotomy, we induced a mid-ventral incision for exposing expose the uterus and intestine. Standard hysterectomy was used for removing the right uterine horns of the animals and it was cut into 4mm^2^ square pieces. We used single 6–0 nylon and 3–0 silk sutures for suturing the serosa layer and abdomen, respectively.

### Grouping and the treatments

In the next step, the animals were assigned into 4 groups including Endo-sole (Endo), Endo-induced rats treated with 3000 and 6000 µg/kg rutin (Rtn-3000 and 6000) and Endo-induced rats treated with vitamin C (0.2 mg/kg) (Vit C) groups. The rats in Endo-sole group received 0.5 mL of saline-normal. Vitamin C was considered as gold standard and administrated as reported by previous studies^30^. Rtn and Vit C were orally administrated for 28 days. This period was selected on the basis of essential time for assessing ameliorative effect of different chemicals against endometriotic-like legions establishment in previous studies^31^.

### Protein extraction and Western Blot

Following test termination (28 days), the rats were euthanized by CO2 gas using a special device. We isolated total protein from endometriotic cells and immunoblotting**/**western blotting performed as described previously^32^. The cells were harvested using 1% Trypsin–EDTA and pelleted. The cell lysates were sonicated in sonication buffer that contained 20 mM Tris–Hcl, 0.5 mM EDTA, 100 mM DEDTC, 1% Tween, 1 mM phenylmethylsulfonyl fluoride, and protease inhibitor cocktail tablets: complete EDTA-free (1 tablet/50 ml) and PhosStop (1 tablet /10 ml). Protein concentration was assessed by Bradford method^33^. Western blot analyses were conducted as reported by previous studies^34^. The analyses were conducted in an amount of protein (50 μg) and samples were loaded and resolved using 12% SDS-PAGE. To prevent nonspecific binding, the membranes were blocked in 5% BSA containing buffer for 2 h at room temperature. These were incubated overnight with the desired primary antibody at its respective dilution at 4 °C. In next step, the membranes were washed by wash buffer TBST (50 mM/L Tris–HCl, pH 7.6, 150 mM/L NaCl, 0.1%Tween 20) and incubated at room temperature for 2 h with appropriate HRP-conjugated secondary antibody (1:15,000 dilution). Results are expressed in relative fold change compared to control (vehicle 1 h). The uncut gel images are presented in Supplementary Data.

### Assessment of apoptosis using TUNEL staining

TUNEL (terminal deoxynucleotidyl transferase enzyme mediated dUTP nick end labeling) Staining assay kit (Roche, Germany) was used for evaluating of the apoptosis ratio as reported by Labat-Moleur et al.,^35^. Summary, xylene (3 changes, each change 5 min) was used for deparaffinization of the Sects. (5 μm) and then rehydrated in graded alcohol (each 2 min). In the next step, the sections were incubated with 1 μL proteinase K for 20 min and washed triplicate by PBS. The sections were then incubated by 5 μL TUNEL solution for 40 min, and washed 3 times with PBS and finally incubated by 10 μL POD-convertor for 0.5 h. The slides were washed triplicate in PBS, incubated with 10 μL DAB substrate for 1 h. The slides were then washed with distilled water. Hematoxylin was used for counting sections and then dehydrated by ascending alcohol. Cells were considered as apoptotic, if cells were observed as clear and dark brown.

### Determination of lipid peroxidation and total antioxidant capacity (TAC)

End product of lipid peroxidation or malondialdehyde (MDA) was investigated for measurement of lipid peroxidation by the absorbance at 535 nm. TAC, SOD and GPx were assessed spectrophotometrically on the basis of procedure described by Rashid et al.^36^.

### Statistical analysis

The results were reported as mean data (± SD). Statistical analysis was performed by the means of one-way analysis of variance (ANOVA), and Tukey test was undertaken for comparing the group means. A P-value less than 0.05 was considered as significant. The data were analyzed for 6 rats/group.

## Supplementary Information

Supplementary Information

## Abbreviations

Bcl-2: B-cell lymphoma protein 2
BAX: BCL2 associated X
DSB: Double strand breaks
GSH: Glutathione
Endo: Endometriosis
MDA: Malondialdehyde
mTOR: Mammalian target of rapamycin
PARP-1: Poly ADP-ribose polymerase-1
ROS: Reactive oxygen species
Rtn: Rutin
TAC: Total antioxidant capacity
TUNEL: Terminal deoxynucleotidyl transferase enzyme mediated dUTP nick end labeling
vitamin c: Vit C: vitamin C
SOD: Super oxide dismutase
GPx: Gutathione peroxidase

## References

[1] Jouhari S, Mohammadzadeh A, Soltanghoraee H, Mohammadi Z, Khazali S, Mirzadegan E, Lakpour N, Fatemi F, Zafardoust S, Mohazzab A, Naderi MM (2018). Effects of silymarin, cabergoline and letrozole on rat model of endometriosis. Taiwanese J. Obstet. Gyne..

[2] Fritzer N, Tammaa A, Haas D, Oppelt P, Renner S, Hornung D, Wölfler M, Ulrich U, Hudelist G (2016). When sex is not on fire: a prospective multicentre study evaluating the short-term effects of radical resection of endometriosis on quality of sex life and dyspareunia. Eur. J. Obstet. Gynecol. Reprod. Biol..

[3] Signorile PG, Baldi A (2010). Endometriosis: new concepts in the pathogenesis. Int. J. Biochem. Cell Biol..

[4] Rogers-Broadway KR, Kumar J, Sisu C, Wander G, Mazey E, Jeyaneethi J, Pados G, Tsolakidis D, Klonos E, Grunt T, Hall M, Chatterjee J, Karteris E (2019). Differential expression of mTOR components in endometriosis and ovarian cancer: effects of rapalogues and dual kinase inhibitors on mTORC1 and mTORC2 stoichiometry. Int. J. Mol. Med..

[5] Tischner D, Woess C, Ottina E, Villunger A (2010). Bcl-2-regulated cell death signaling in the prevention of autoimmunity. Cell. Death Dis..

[6] Kulsoom B, Shamsi TS, Afsar NA, Memon Z, Ahmed N, Hasnain SN (2018). Bax, Bcl-2, and Bax/Bcl-2 as prognostic markers in acute myeloid leukemia: are we ready for Bcl-2-directed therapy?. Cancer Manag. Res..

[7] Zhang H, Zhao X, Liu S, Li J, Wen Z, Li M (2010). 17βE2 promotes cell proliferation in endometriosis by decreasing PTEN via NFκB-dependent pathway. Mol. Cell. Endocrinol..

[8] Satoh MS, Lindahl T (1992). Role of poly(ADP-ribose) formation in DNA repair. Nature.

[9] Philip CA, Laskov I, Beauchamp MC, Marques M, Amin O, Bitharas J, Kessous R, Kogan L, Baloch T, Gotlieb WH, Yasmeen A (2017). Inhibition of PI3K-AKT-mTOR pathway sensitizes endometrial cancer cell lines to PARP inhibitors. BMC Cancer.

[10] Harada T, Taniguchi F, Izawa M, Ohama Y, Takenaka Y, Tagashira Y, Ikeda A, Watanabe A, Iwabe T, Terakawa N (2007). Apoptosis and endometriosis. Front. Biosci..

[11] Scutiero G, Iannone P, Bernardi G, Bonaccorsi G, Spadaro S, Volta CA, Greco P, Nappi L (2017). Oxidative stress and endometriosis: A systematic review of the literature. Oxid. Med. Cell. Longev..

[12] Agustin-Salazar S, Medina-Juarez LA, Soto-Valdez H, Manzanares-Lopez F, Gamez-Meza N (2014). Influence of the solvent system on the composition of phenolic substances and antioxidant capacity of extracts of grape (*Vitis vinifera L.*) marc. Aust. J. Grape Wine Res..

[13] Cruz-Zúñiga JM, Soto-Valdez H, Peralta E, Mendoza-Wilson AM, Robles-Burgueño MR, Auras R, Gámez-Meza N (2016). Development of an antioxidant biomaterial by promoting the deglycosylation of rutin to isoquercetin and quercetin. Food Chem..

[14] Ananth DA, Rameshkumar A, Jeyadevi R, Jagadeeswari S, Nagarajan N, Renganathan R (2015). Antibacterial potential of rutin conjugated with thioglycolic acid cappedcadmiumtelluridequantumdots (TGACdTe QDs). Spectrochim. Acta A..

[15] Almutairi MM, Alanazi WA, Alshammari MA, Alotaibi MR, Alhoshani AR, Al-Rejaie SS (2017). Neuro-protective effect of rutin against Cisplatin-induced neurotoxic rat model. BMC Complement. Altern. Med..

[16] Butchi Akondi R, Kumar P, Annapurna A, Pujari M (2011). Protective effect of rutin and naringin on sperm quality in streptozotocin (STZ) induced type 1 diabetic rats. Iran. J. Pharm. Res..

[17] Elsayed HE, Ebrahim HY, Mohyeldin MM, Siddique AB, Kamal AM, Haggag EG, El Sayed KA (2017). Rutin as a novel c-met inhibitory lead for the control of triple negative breast malignancies. Nutr. Cancer..

[18] Vadapalli U, Muvvala S, Alluri R, Lakshmi BVS (2017). Antiproliferative activity of rutin on HeLa cell line induced cervical cancer in rats. Int. J. Pharm. Sci. Rev. Res..

[19] Harlev A, Gupta S, Agarwal A (2015). Targeting oxidative stress to treat endometriosis. Expert Opin. Ther. Targets..

[20] Rosa e Silva JC, do Amara VF, Mendonça JL, Rosa e Silva AC, Nakao LS, Poli Neto OB, Ferriani RA (2014). Serum markers of oxidative stress and endometriosis. Clin. Exp. Obstet. Gynecol..

[21] McLaren J, Prentice A, Charnock-Jones DS, Sharkey AM, Smith SK (1997). Immunolocalization of the apoptosis regulating proteins Bcl-2 and Bax in human endometrium and isolated peritoneal fluid macrophages. Hum. Reprod..

[22] Katkoori VR, Suarez-Cuervo C, Shanmugam C, Jhala NC, Callens T, Messiaen L, Posey J, Bumpers HL, Meleth S, Grizzle WE, Manne U (2010). Bax expression is a candidate prognostic and predictive marker of colorectal cancer. J. Gastrointest. Oncol..

[23] Singh L, Pushker N, Saini N, Sen S, Sharma A, Bakhshi S, Chawla B, Kashyap S (2015). Expression of pro-apoptotic Bax and anti-apoptotic Bcl-2 proteins in human retinoblastoma. Clin. Exp. Ophthalmol..

[24] Aksu EH, Kandemir FM, Özkaraca M, Ömür AD, Küçükler S, Çomaklı S (2017). Rutin ameliorates cisplatin-induced reproductive damage via suppression of oxidative stress and apoptosis in adult male rats. Andrologia.

[25] Cho YJ, Lee SH, Park JW, Han M, Park MJ, Han SJ (2018). Dysfunctional signaling underlying endometriosis: current state of knowledge. J. Mol. Endocrinol..

[26] Cinar O, Seval Y, Uz YH, Cakmak H, Ulukus M, Kayisli UA, Arici A (2009). Differential regulation of Akt phosphorylation in endometriosis. Reprod. Biomed. Online.

[27] Leconte M, Nicco C, Ngô C, Chéreau C, Chouzenoux S, Marut W, Guibourdenche J, Arkwright S, Weill B, Chapron C, Dousset B (2011). The mTOR/AKT inhibitor temsirolimus prevents deep infiltrating endometriosis in mice. Am. J. Pathol..

[28] Gupta S, Agarwal A, Krajcir N, Alvarez JG (2006). Role of oxidative stress in endometriosis. Reprod. Biomed. Online..

[29] Bilotas M, Meresman G, Stella I, Sueldo C, Baranao RI (2010). Effect of aromatase inhibitors on ectopic endometrial growth and peritoneal environment in a mouse model of endometriosis. Fertil. Steril..

[30] Laili AN, Ananingsih I, Wiyasa IWA, Indrawan IWA, Barlianto W, Yueniwati Y (2015). Protective effect of combined vitamin C and E against ovarian and endometrial toxicity in rats that receiving oral rhodamine B. Biomark. Genomic Med..

[31] Jin, Z., Wang, L., & Zhu, Z. Effect of GuiXiong Xiaoyi Wan in treatment of endometriosis on rats. Evid. Complement. Alternat. Med. 208514 (2015). 10.1155/2015/208514 PMC4322821 25691906

[32] Arosh JA, Banu SK (2019). Dual inhibition of ERK1/2 and AKT pathways is required to suppress the growth and survival of endometriotic cells and lesions. Mol. Cell. Endocrinol..

[33] Bradford MM (1976). A rapid and sensitive method for the quantitation of microgram quantities of protein utilizing the principle of protein-dye binding. Anal. Biochem..

[34] Saha S, Sadhukhan P, Sinha K, Agarwal N, Sil PC (2016). Mangiferin attenuates oxidative stress induced renal cell damage through activation of PI3K induced Akt and Nrf-2 mediated signaling pathways. Biochem. Biophys. Rep..

[35] Labat-Moleur F, Guillermet C, Lorimier P, Robert C, Lantuejoul S, Brambilla E, Negoescu A (1998). TUNEL apoptotic cell detection in tissue sections: critical evaluation and improvement. J. Histochem. Cytochem..

[36] Rashid K, Das J, Sil PC (2013). Taurine ameliorate alloxan induced oxidative stress and intrinsic apoptotic pathway in the hepatic tissue of diabetic rats. Food Chem. Toxicol..

