# Supplementary material for: The effectiveness of Rutin for prevention of surgical induced endometriosis development in a rat model
Source: Sci Rep. 2021 Mar 30;11:7180. doi: 10.1038/s41598-021-86586-4 (PMC8010059; doi:10.1038/s41598-021-86586-4)
Supplement: Supplementary file 1 — Supplementary Information [file 41598_2021_86586_MOESM1_ESM.pdf]

Supporting Information to

The effectiveness of *Rutin* for prevention of surgical induced endometriosis development in a rat model

Hatef Talebi [a], Mohammad Reza Farahpour *[b], Hamed Hamishehkar [c]

[a] Department of Basic Sciences, Faculty of Veterinary Medicine, Urmia Branch, Islamic Azad University, Urmia, Iran.

[b] Department of Clinical Sciences, Faculty of Veterinary Medicine, Urmia Branch, Islamic Azad University, Urmia, Iran.

[c] Drug Applied Research Center, Tabriz University of Medical Sciences, Tabriz, Iran.

***Correspondence to:** Mohammad Reza Farahpour, D.V.M., D.V. Sc., Department of Clinical Sciences, Faculty of Veterinary Medicine, Urmia Branch, Islamic Azad University, Urmia, 57159-44867, Iran. Tel: +98 4414373676; Fax: +98 4433460980. E-mail: mrf78s@gmail.com. ORCID ID: orcid.org/0000-0001-8631-071X

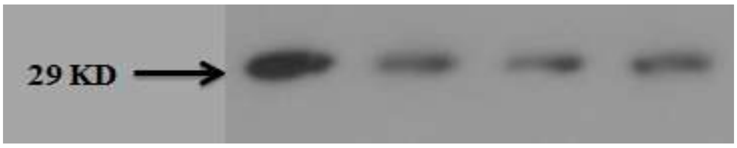

Figure 1: Full length gel of western blot for BCL2.

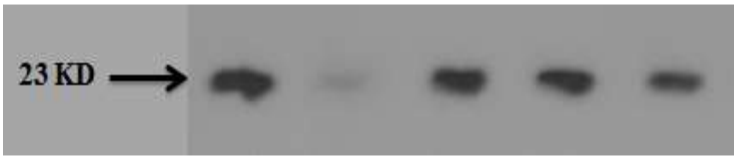

Figure 1: Full length gel of western blot for BAX.

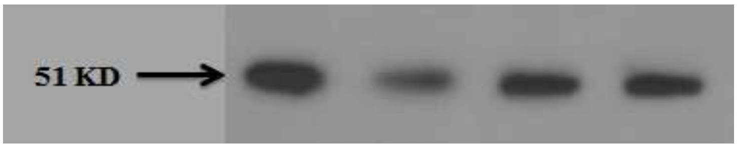

Figure 1: Full length gel of western blot for Caspase9.

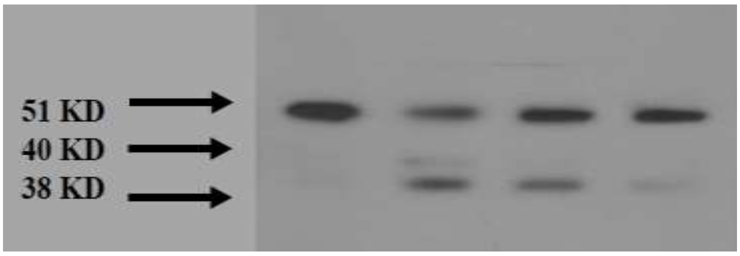

Figure 1: Full length gel of western blot for Procaspase9 and Cleaved caspase9.

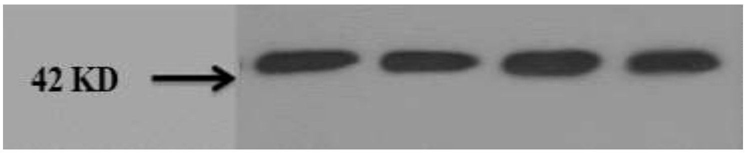

Figure 1: Full length gel of western blot for β-Actin.

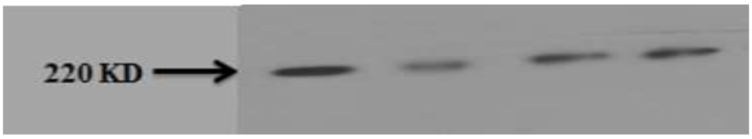

Figure 2: Full length gel of western blot for p-mTOR.

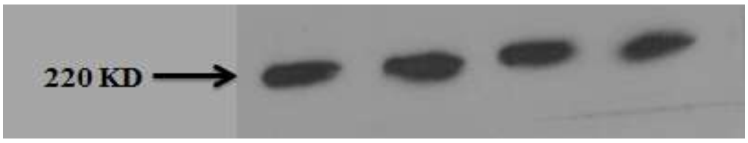

Figure 2: Full length gel of western blot for mTOR.

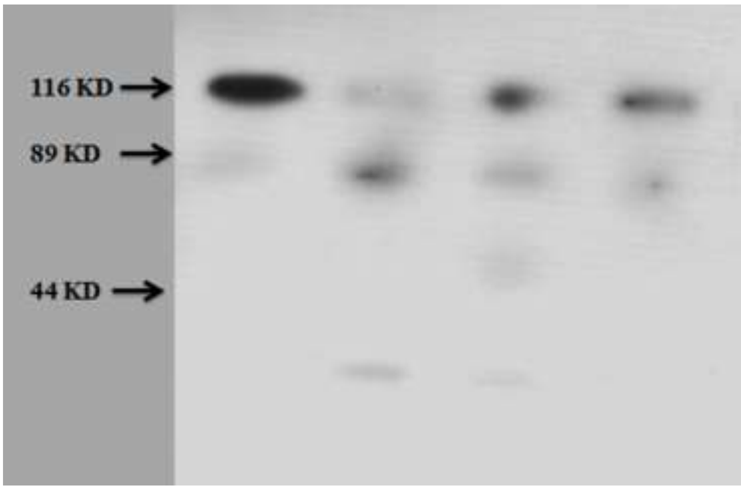

Figure 2: Full length gel of western blot for PARP (pro) and cleaved PARP.

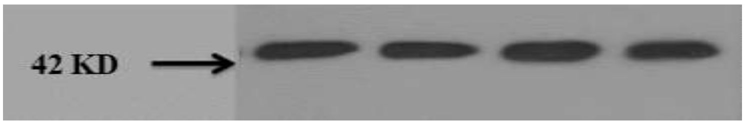

Figure 2: Full length gel of western blot for β-Actin.
